# Effects of collective volleyball cooperative tactical training on social anxiety and peer interaction ability in school-aged children: a controlled intervention study

**DOI:** 10.3389/fpubh.2026.1952919

**Published:** 2026-10-28

**Authors:** Qiaoxian Zhang, Yuli Zhou

**Affiliations:** 1 School of Competitive Sports, Shandong Sport University, Rizhao, China; 2 Unit 32128 of the People's Liberation Army of China, Jinan, China

**Keywords:** cooperative tactical training, intervention study, peer interaction ability, physical education, school-aged children, social anxiety, volleyball

## Abstract

**Background:**

School-aged children that experience social anxiety and have difficulty with interactions among peers can create significant challenges to their development in terms of both psychology and behavior. A team sport such as volleyball provides a common setting that naturally includes all aspects of social interaction (communication, cooperation, role coordination, etc.) and shared goals which is essential to improve the child’s confidence in interacting socially and abilities to interact socially with peers.

**Objective:**

This research is an examination of the effect that a 12-week collective volleyball cooperative tactical training program will have on social anxiety and peer interaction in children (8–12).

**Methods:**

A sample of 80 healthy school-age children was obtained via two methods of campus based, voluntary and/or open recruitment. These children were then randomly assigned to either an experimental intervention group (*n* = 40) or an active control group receiving standard physical education (*n* = 40). For 12 consecutive weeks, participants in the experimental group engaged in a type of volleyball cooperative tactical team training that occurred on 3 days each week at 1 h per day. During this time the participants in the control group engaged in standard physical education classes of matched frequency and duration. Social anxiety was measured by use of the Social Anxiety Scale for Children (SASC); SASC measures include total social anxiety, fear of negative evaluation and social avoidance/distress. Participant peer interaction was evaluated using a study-specific 25-item peer-interaction questionnaire covering interaction initiative, peer acceptance, interaction coordination, interaction openness, and conflict resolution ability. Data collection occurred prior to, as well as subsequent to, the intervention. Statistical analyses were conducted utilizing SPSS version 26.0 software. A 2 × 2 mixed-design ANOVA was conducted to examine Group × Time interaction effects, with group (intervention vs. control) as the between-subjects factor and time (pre-intervention vs. post-intervention) as the within-subject factor. Effect sizes were reported using partial eta squared (*η*^2^*p*), and *post hoc* comparisons were performed when significant interactions were identified.

**Results:**

Group × Time Interaction Effects from a mixed-design ANOVA were found to be statistically significant for both of the outcomes (social anxiety and peer interaction) of this study. Children participating in an intervention that included an instructional program using the Collective Volleyball Cooperative Tactical Training Model showed significantly greater improvement than did those in the Control Condition in reducing their level of social anxiety as well as improving their peer interaction abilities over the course of the 12-weeks. Although we found a statistically-significant improvement in both children’s social anxiety, and their peer interaction skills through a 12-week program, there is no data available for us to understand if those positive results will continue into the future because we did not assess children at later points in time. Thus, future researchers are encouraged to consider extended assessment periods as part of their study designs to determine how long such interventions have an impact. Secondly, as a feasibility study in a controlled environment showed promise for implementation of cooperative tactical volleyball programs, there may be barriers to successful implementation in regular school settings such as lack of facilities, class size and teacher workload. Future research is needed to examine how cooperative tactical volleyball programs can be adapted to encompass large groups of students and incorporated into routine physical education curriculum. Furthermore, although the intervention showed feasibility under controlled conditions, implementation in regular school settings may face practical challenges, including limited facilities, larger class sizes, and teacher workload. Future studies should examine how cooperative volleyball tactical training can be adapted for broader school-based implementation.

**Conclusion:**

Collective Volleyball Cooperative Tactical Training is an effective method for reducing social anxiety and improving the ability of school aged children to interact with peers. The program offers a viable, school-based model for integrating sport skills acquisition (training) and psychosocial growth through physical activity.

## 1 Introduction

Children in the ages of eight through twelve have opportunities that allow them to develop into emotionally intelligent individuals, socially aware as it relates to other people and themselves. School-age children begin to develop their social connections; they move from being family centered in many of their social experiences to developing peer-to-peer relationships and participating with peers. Positive peer interactions will aid children in developing an understanding of how others feel, develop cooperative behavior, build confidence and become competent socially. Negative peer interaction experiences can lead to problems for children at school, for their mental health and future social development (1–4).

Children with high levels of social anxiety can be reluctant to join in on group activities. They tend to communicate less actively. Many avoid new social environments because they worry about being wrong in front of their peers. Social anxiety is one of many issues that can negatively affect how well students form friendships. Students who experience social anxiety will also have difficulty participating in class. The anxiety can further contribute to their emotional imbalance and lower confidence (5–8).

Peer interaction skills are also an important aspect of assessing children’s social development. Peer interaction abilities reflect a child’s ability to start interactions, have peers accept them as friends, have peers want to be around them, feel comfortable expressing themselves to others, and have conflict resolution skills when interacting socially. Children who develop strong peer interaction skills can form long lasting friendships, work together on group projects, and adjust well to the demands of school. Children who struggle with developing strong peer interaction skills will likely exhibit lower levels of initiating conversations, lower levels of cooperative behaviors, less acceptance by their peers, and difficulty resolving conflicts which in turn could lead to feelings of isolation or social anxiety and create a cycle of ongoing social discomfort and anxiety related to social situations (3, 4, 9, 10).

Children’s possibilities of engaging with their peers through face-to-face interactions are being negatively impacted because of increased pressures to succeed academically, less time spent outdoors, and more time using electronic technology. Increasingly, there is concern about a growing number of school aged children who withdraw socially, display passive interpersonal behaviors and experience difficulties communicating with peers. Thus developing accessible, organized and youth friendly strategies that can be used as interventions in schools, while promoting child mental health has become *a priori*ty area in both the development of school based physical education and child mental health prevention (10–12).

Physical activity is a way to promote both physical development and social/psychological development. Physical activity provides more experience-based, interactive opportunities for children than do most other forms of verbal-only intervention. Furthermore, team sports provide children with opportunities to work together in order to accomplish common goals. When children play on teams they need to interact with each other; they need to be able to read what their teammates want them to do next; they will have to adapt their own behaviors based upon what their teammates are doing so that the team can meet its goals; and when working together they will learn how to successfully achieve a goal. Therefore, all of these experiences are very similar to the ways children will interact socially throughout life.

Team sports for children who are socially anxious can be an effective way of developing relationships at a lower level of stress compared to other group settings. The interaction that occurs as a result of teamwork is generally based on physical activity, therefore it does not require formal verbal exchange. Therefore, the child’s stress related to social judgments is reduced. As the child works successfully in groups over time, he or she will become more confident interacting with others, avoid being involved in social situations less often and have more desire to take part in group work (6, 13–15).

Volleyball is one of many team sports which emphasize teamwork, communication, and coordination. The skills of passing, receiving, setting, attacking, and defense cannot be accomplished as separate and independent acts on the part of the player; these are all interdependent and require the coordinated efforts of the team. In order to participate in volleyball, school-aged children will have opportunities to view their teammate’s position(s) and respond to a variety of different playing conditions. Young players will also have the opportunity to express verbally or through body language their own intentions related to strategy. Furthermore, young players will have the opportunity to assist their teammates at times when they can most benefit from such help. All of these elements combine to make volleyball a suitable vehicle for the development of social skills that allow children to interact with each other as peers.

Traditional volleyball training is based primarily on a player’s ability to perform technically correct skills. The primary focus in traditional volleyball training is on the individual performing their specific role or position. Cooperative tactical volleyball training focuses on the collaboration among teammates as well as developing strategies for working together effectively as a team. Players will be required to collaborate with each other; they will be challenged to work with their teammate(s) and/or small group; they will have to develop strategies to accomplish tasks collectively; and they will have to develop trust by participating in the same sport activities (16–20).

While research has demonstrated that sport involvement can positively influence child/adolescent psychological and social development, there are still several restrictions to this area. Most studies measure the impact of “general” or “unstructured” physical activity participation, while few have examined the effect of structurally designed team-sport based interventions for developing social interaction and reducing social anxiety. School physical education programs typically concentrate on developing student’s physical skills and fitness levels; however, the psychological and social aspects of using sport as an educational tool are rarely developed in a systematic manner. Volleyball is one of the most accessible team sports played by students in elementary through high school, with numerous cooperative features. However, empirical support for the use of volleyball as a cooperative tactic based on a team training environment as a psychosocial intervention program is very limited (11, 14, 17, 21).

The purpose of this study was to determine how participating in a 12-week collective volleyball cooperative tactical training program could affect social anxiety and improve a child’s ability to interact with their peers who are also children (ages 8–12). The researchers predicted that children receiving the volleyball cooperative tactical intervention would have less social anxiety, and be able to better interact with other children compared to children who participated in standard physical education.

## 2 Methods

### 2.1 Study design and ethical considerations

In this investigation we used a controlled intervention study to examine whether a volleyball cooperative tactical group training program could reduce social anxiety and improve peer interaction skills in children. A review of the study’s protocol was performed by the Ethics Review Board of the School of Competitive Sports, Shandong Sport University, China (approval number 2026089), all procedures of which were carried out in accordance with the Ethical Principles for Researching Human Participants and the Declaration of Helsinki.

Children’s participation in this study was completely voluntary; they could withdraw from the study for any reason at any point without fear of reprisal. Children along with their parents/guardians were given a full explanation of the reasons behind this study, the methods used in the study, potential dangers to the child (if any), how confidential the information would be, and that the children had an unconditional right to remove themselves from the study if they wanted to do so.

All of the intervention activities were designed with a low-intensity, progressive design that would support both physically and psychologically developing children. The professional Physical Education Teachers supervised all aspects of the child’s training. Prior to each session a structured Warm-Up activity was completed by all participants; Stretching and Relaxation were incorporated as post-activity exercises to aid in reducing Fatigue and Injury Risk (21, 22).

### 2.2 Participants

A total of eighty (80) healthy school-aged students participated voluntarily or as part of their family’s mandatory school registration process. The participants were randomly divided into two groups; an experimental group with forty (40) students and a control group with forty (40) students.

The selection criteria were as follows: Children (aged 8–12 years); Physically healthy and mentally healthy; No significant physical illness, or an exercise contraindication, or a diagnosis of a psychiatric disorder; Voluntary to participate with signed written informed consent from the child’s legal guardian; Not participating regularly in a previously trained special team sport.

Excluded were children with a diagnosis of either heart disease, asthma, bone/joint injuries or other medical reasons that would prohibit them from exercising; children who have been formally diagnosed as having an Autism Spectrum Disorder, Anxiety Disorders, Depression or other Psychiatric Disorders; and those participating in Long-Term Team Sports Training for Volleyball, Basketball, etc.; and those unable to participate through the entire Intervention Cycle and Data Collection Process.

### 2.3 Group allocation

After completing an initial eligibility screening and a baseline assessment of each participant, they were randomly assigned to one of two groups. Participants who were part of the experimental group received Collective Volleyball Cooperative Tactical Training (CVC), while participants who were members of the Control Group, as a comparison condition to Experimental Group members, participated in standard physical education with no CVC.

After participants were screened for eligibility and completed baseline assessment, they were randomly assigned to either the Experimental Group or the Control Group using a computer-generated randomization sequence. The sequence was generated by an independent researcher who was not involved in intervention delivery. To maintain allocation concealment, group assignments were placed in sealed opaque envelopes prepared by the independent researcher. The envelopes remained closed until the participant had completed the baseline assessment and were opened only at the point of assignment. Thus, the intervention team did not know the upcoming allocation before baseline data collection was completed.

Baseline demographic characteristics and primary outcome measures were compared before intervention to confirm comparability between groups.

### 2.4 Intervention protocol

The 12-week-long intervention was structured as follows. Participants in the experimental group received 3×60 minute collective volleyball cooperative tactical training per week. All training sessions were supervised by qualified PE-teachers.

The intervention was divided into three progressive stages (Figure 1).

**Figure 1:**
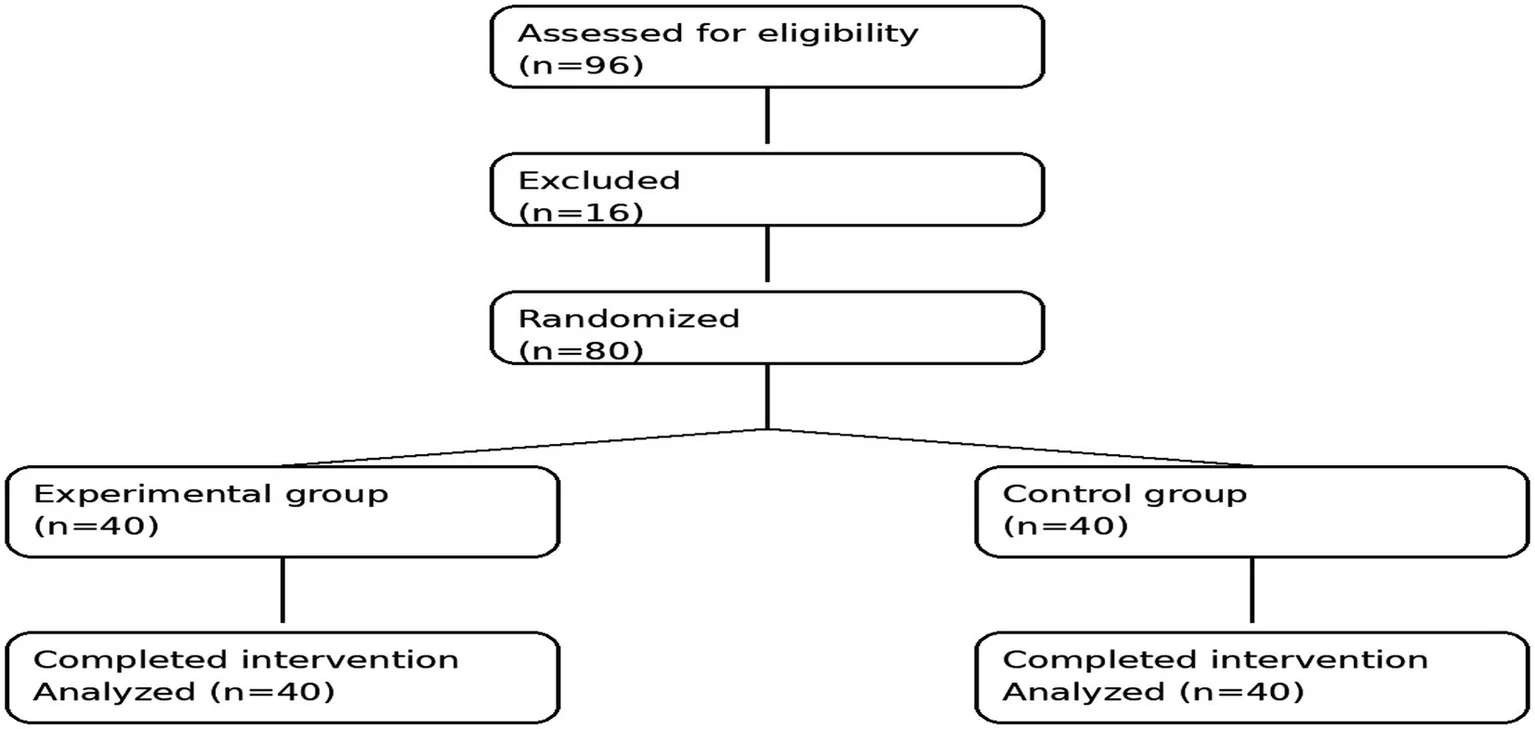
CONSORT flow diagram of participant recruitment, allocation, intervention, and analysis.

Weeks 1–2 were based on a phase of basic adaptation. All of the children performed basic skills for Volleyball such as basic movement skills and other simple team-based activities. This phase helped to get all of the children comfortable in their new environment so that they would be able to work together and create an early sense of team cooperation.

All coaches had more than 5 years of physical education coaching experience as a certified instructor. Instructors also completed the same standard training prior to conducting the intervention on both cooperative tactical strategies and psychological objectives in order for all instructors to have the same knowledge of how to conduct the intervention consistently.

From Week 3 to Week 10 of the study, the interventions moved into the core tactical training phase for co-op play in a multi-player environment. The primary focus during this period was on two players working together with each other through their passes and receives. Additionally, the interventions were based upon a large group (team) that would work cooperatively as an offense or defense. It also involved coordinating all roles of players and completing tasks within groups. During this time, there was a great emphasis placed upon developing and maintaining open lines of communication amongst teammates; working together as a unit; supporting one another while in a game situation; and overall coordinated effort as a team.

Consolidation Training and Post-Intervention Assessment occurred during weeks 11 and 12 of the study. The children practiced and refined their newly developed cooperative tactics that they had previously learned through prior sessions. Data Collection for this phase ended at week 12 the Control Group took part in the usual Physical Education Program for the same time frame as the Experimental Group. This consisted of standard physical exercise and typical school sports programs, however these programs did NOT include a structure to facilitate volleyball cooperative tactics, volleyball team tactic work together, or formal peer based task coordination.

The Control Group had the same amount of Physical Education participation as did the Experimental Group (same number of hours and times a week) that allowed them to have the same opportunity for physical activity and Instructor Contact Time. However, they were excluded from the Cooperative Tactical Volleyball portions. This was done to limit other variables that could be attributed to an unequal amount of Exercise Volume and Researcher Attention.

### 2.5 Outcome measures

Outcome assessments were conducted before the intervention and after the 12-week intervention.

Total social anxiety, and fear of being evaluated negatively by others and how much social avoidance/distress are all measured on the Social Anxiety Scale for Children (SASC), which is scored from 0 to 52; the higher your total SASC score, the greater your social anxiety is (5).

Peer interaction ability was assessed using a study-specific 25-item Peer Interaction Ability Questionnaire developed for the present study. The measurement rationale was informed by the Interpersonal Competence Questionnaire (ICQ) framework introduced by Buhrmester et al. (23), which distinguishes multiple domains of interpersonal competence in peer relationships. Subsequent psychometric work in Chinese adolescents has supported the reliability and five-factor structure of a brief ICQ adaptation (ICQ-15) (24), and a Chinese youth study has reported the use of a 25-item, five-domain shortened ICQ form with five items per domain and an overall Cronbach’s *α* of 0.91 (25). These studies provide an external conceptual and psychometric basis for a multidimensional interpersonal-competence framework and demonstrate the feasibility of brief adaptations in Chinese populations; however, they do not constitute external validation of the exact 25-item questionnaire used in the present study. The questionnaire used here operationalized five study-specific peer-interaction domains—interaction initiative, peer acceptance, interaction coordination, interaction openness, and conflict resolution ability—with five items in each domain. The domain content was additionally informed by the child peer-relations and youth-sport peer-functioning literature already cited in the manuscript (3, 4, 15, 19). Each item was rated on a 5-point Likert scale from 1 (strongly disagree) to 5 (strongly agree), with higher scores indicating stronger peer interaction ability. Each dimension score was calculated as the arithmetic mean of its five corresponding items and analyzed separately. Because the exact item set and domain labels used in this study differ from the published ICQ and its validated brief adaptations, we do not characterize the present questionnaire as a previously validated standardized instrument. Internal consistency for the complete 25-item questionnaire in the present sample was Cronbach’s *α* = 0.91; this coefficient is reported as sample-specific reliability evidence and should not be interpreted as establishing full construct validity. Full structural and scoring details are provided in Supplementary Table S2.

### 2.6 Safety monitoring

The current investigation represented an individually tailored, low risk intervention of physical activity. Each participant prior to participating in a training session would be required to complete a series of warm up movements. Following completion of the training session each child participated in a series of stretching and relaxing activities. Participants’ levels of physiological development influenced both the duration and intensity at which their training sessions occurred (21, 22).

Teachers checked during the intervention to ensure that each participant’s body (physical) and mind (emotional), as well as their response to the training process. Training would be halted if a participant exhibited signs of fatigue, mild muscle soreness, emotional resistance or discomfort. In addition, in cases of minor injuries sustained through sport, emergency first aid treatments could be administered at the training site. Parents/guardians were notified immediately of this incident.

Questionnaires were distributed to students in a quiet classroom by trained research assistants who did not participate in delivering the intervention. Due to the physical nature of the intervention, participants and instructors could not be blinded to the assigned condition. To reduce, although not eliminate, expectancy and social desirability influences, students were informed that questionnaire responses were anonymous, confidential, unrelated to school evaluation, and would not affect their continued participation. The independent research assistants used standardized instructions and did not provide feedback on participants’ answers.

### 2.7 Statistical analysis

Statistical analysis for all statistical tests was completed using SPSS 26.0. The continuous data is represented as mean ± SD; the categorical data is represented as frequency and percentage. To determine if there are differences in how the two groups responded to the intervention (Group × Time), a 2 × 2 mixed ANOVA will be used. This study will use group (Experimental or Control) as the between subjects variable and time (Pre-Intervention vs. Post-Intervention) as the within subject variable. If there is a significant difference at post-test between the experimental and control group the effect size will be calculated as partial *η*^2^*p*. When significant interactions occur, *post hoc* tests will be utilized to identify where the significant differences occurred.

An a priori power calculation was performed in order to determine the sample size needed. Using G*Power 3.1 with an expected moderate effect size (*f* = 0.25), alpha (0.05), and power (0.8) for the repeated measures Group × Time interaction, it was determined that 80 participants were needed.

## 3 Results

### 3.1 Baseline characteristics

The study had a total of 80 school-aged children that were randomly divided into two groups (the experimental group *n* = 40 and the control group *n* = 40). Each participant was able to complete both the baseline evaluation and the 12-week intervention. There were no dropouts due to physical or psychological discomfort, emotional distress, or side effects associated with the interventions.

Prior to the intervention, there were no statistically significant differences between the two groups in age, sex, SASC total score, fear of negative evaluation, social avoidance and distress, or any of the five peer-interaction dimensions (all *p* > 0.05), indicating comparable baseline characteristics (Table 1).

**Table 1:** Baseline characteristics of participants.

Variables	Experimental group (*n* = 40)	Control group (*n* = 40)	*p*-value
Age (years)	10.08 ± 1.22	10.15 ± 1.19	0.796
Gender (Male/Female)	21/19	20/20	0.823
SASC Total Score	31.62 ± 5.14	31.28 ± 5.06	0.766
Fear of Negative Evaluation	12.18 ± 2.36	12.05 ± 2.29	0.803
Social Avoidance and Distress	19.44 ± 3.48	19.23 ± 3.42	0.786
Interaction Initiative	2.83 ± 0.46	2.85 ± 0.44	0.842
Peer Acceptance	2.91 ± 0.42	2.89 ± 0.43	0.834
Interaction Coordination	2.80 ± 0.47	2.83 ± 0.45	0.771
Interaction Openness	2.86 ± 0.44	2.88 ± 0.46	0.843
Conflict Resolution Ability	2.78 ± 0.48	2.82 ± 0.47	0.708

### 3.2 Changes in social anxiety after intervention

A two-way (Group × Time) repeated measures analysis of variance with two levels of time (pre-post) and two levels of group (experimental-control), conducted on total social anxiety scores after completion of the 12-week treatment program, showed an extremely high level of statistical significance for both main effect of Time, *F*(1, 78) = 13.04, *p* = 0.0008 and the Group × Time interaction *F*(1, 78) = 68.42, *p* < 0.0001; partial eta squared = 0.467. In addition to these results, there was a highly statistically significant main effect of Time for Fear of Negative Evaluation *F*(1, 78) = 9.29, *p* = 0.003 and Social Avoidance and Distress *F*(1, 78) = 10.25, *p* = 0.002 and a highly statistically significant Group × Time interaction effect for both Fear of Negative Evaluation *F*(1, 78) = 54.76, *p* < 0.0001; partial eta squared = 0.413 and Social Avoidance and Distress *F*(1, 78) = 61.35, *p* < 0.0001; partial eta squared = 0.440. The significant interaction effects indicated that all participants demonstrated a decrease in their social anxiety symptoms over time but the reductions were larger in magnitude for those who received the active treatment compared to those in the standard physical education control group.

*Post hoc* analyses revealed that compared with children who participated in the standard physical education control condition during the same 12-week study period, children that received volleyball-based cooperative tactical team sports training exhibited larger gains on all of the posttest measures of social anxiety; therefore, it appears as though the specific nature of the program was directly related to its effect on reducing social anxiety.

These study results demonstrate that collective volleyball cooperative team tactics training positively impacted young children’s levels of social anxiety; particularly with respect to their fear of negative evaluation (i.e., social evaluative concerns) and their level of social (Figures 2, 3; Table 2).

**Figure 2:**
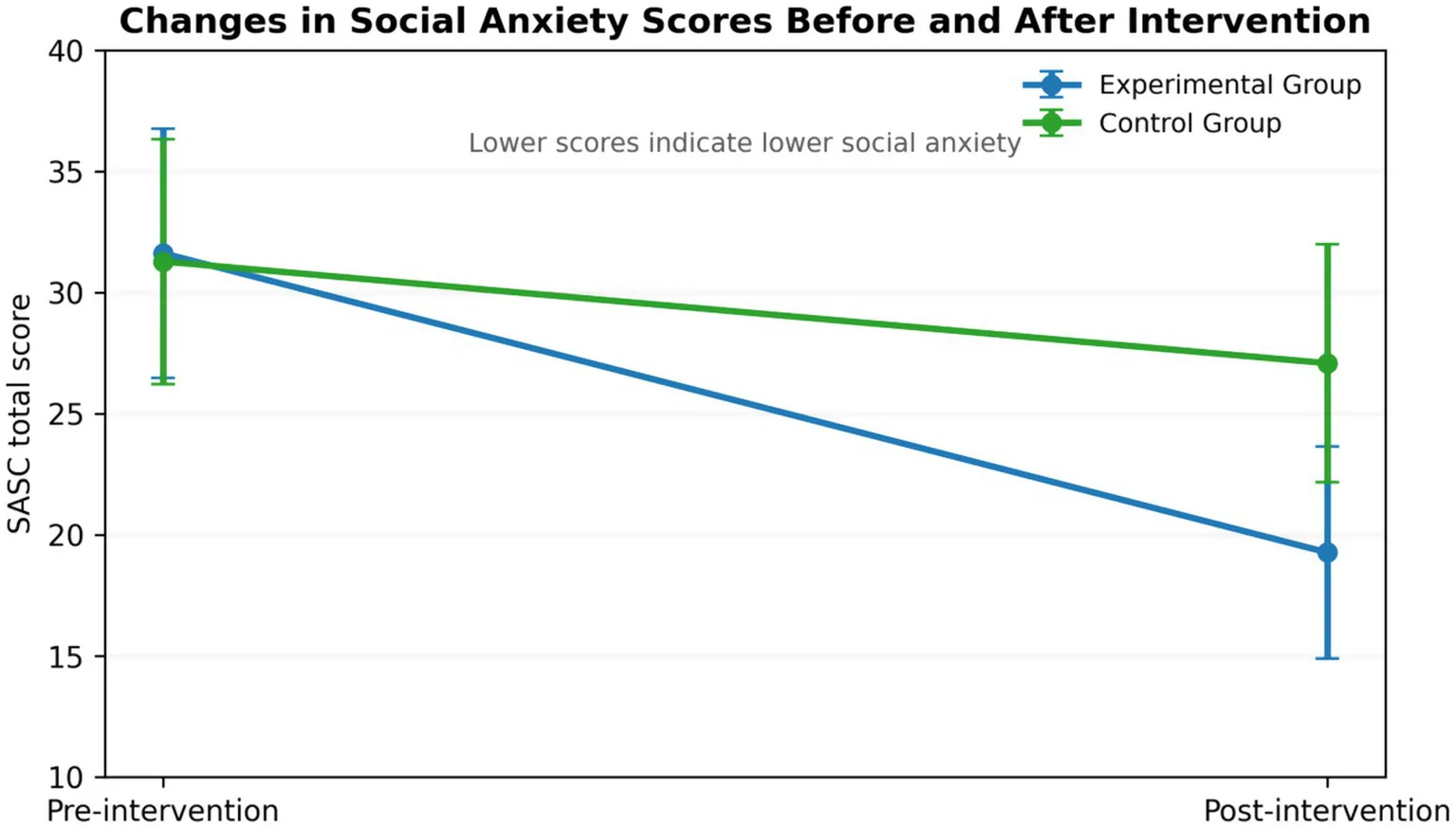
Changes in SASC total scores before and after the 12-week intervention.

**Figure 3:**
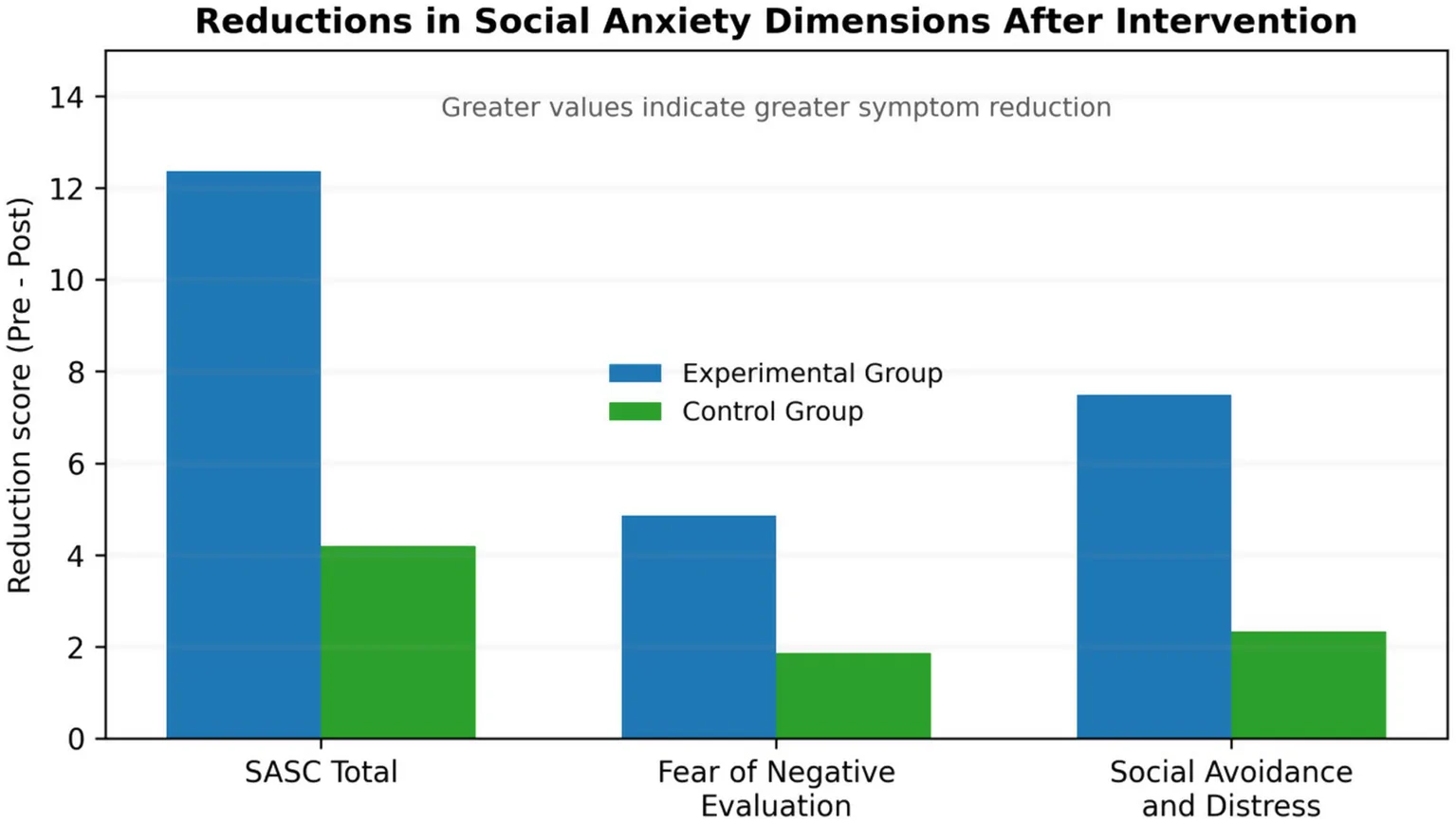
Reductions in social anxiety dimensions after the intervention.

**Table 2:** Descriptive statistics of social anxiety scores before and after intervention.

Variables	Time	Experimental group (*n* = 40)	Control group (*n* = 40)	*p*-value
SASC Total Score	Pre-intervention	31.62 ± 5.14	31.28 ± 5.06	0.766
	Post-intervention	19.26 ± 4.38	27.08 ± 4.91	<0.001
Fear of Negative Evaluation	Pre-intervention	12.18 ± 2.36	12.05 ± 2.29	0.803
	Post-intervention	7.32 ± 1.74	10.18 ± 2.08	<0.001
Social Avoidance and Distress	Pre-intervention	19.44 ± 3.48	19.23 ± 3.42	0.786
	Post-intervention	11.94 ± 3.05	16.90 ± 3.24	<0.001

### 3.3 Changes in peer interaction ability after intervention

Peer interaction ability improved after the intervention, with greater improvement observed in the intervention group. Significant Group × Time effects were identified for all five prespecified peer-interaction dimensions: interaction initiative, peer acceptance, interaction coordination, interaction openness, and conflict resolution ability.

Descriptive comparisons showed that scores on each of the five peer-interaction dimensions increased from pre- to post-intervention in the experimental group. Although modest improvements were also observed in the standard physical education control group, post-intervention scores were higher in the experimental group across all five dimensions.

These findings suggest that the structured volleyball cooperative tactical training will improve young children’s peer interaction skills through repeated opportunities to communicate with each other, work together, coordinate roles, and resolve conflicts while (Table 3; Figure 4).

**Table 3:** Descriptive statistics of peer interaction ability before and after intervention.

Variables	Time	Experimental group (*n* = 40)	Control group (*n* = 40)	*p*-value
Interaction Initiative	Pre-intervention	2.83 ± 0.46	2.85 ± 0.44	0.842
	Post-intervention	4.02 ± 0.42	3.30 ± 0.44	<0.001
Peer Acceptance	Pre-intervention	2.91 ± 0.42	2.89 ± 0.43	0.834
	Post-intervention	4.06 ± 0.39	3.35 ± 0.43	<0.001
Interaction Coordination	Pre-intervention	2.80 ± 0.47	2.83 ± 0.45	0.771
	Post-intervention	3.95 ± 0.41	3.27 ± 0.45	<0.001
Interaction Openness	Pre-intervention	2.86 ± 0.44	2.88 ± 0.46	0.843
	Post-intervention	3.91 ± 0.40	3.25 ± 0.46	<0.001
Conflict Resolution Ability	Pre-intervention	2.78 ± 0.48	2.82 ± 0.47	0.708
	Post-intervention	3.86 ± 0.43	3.18 ± 0.48	<0.001

**Figure 4:**
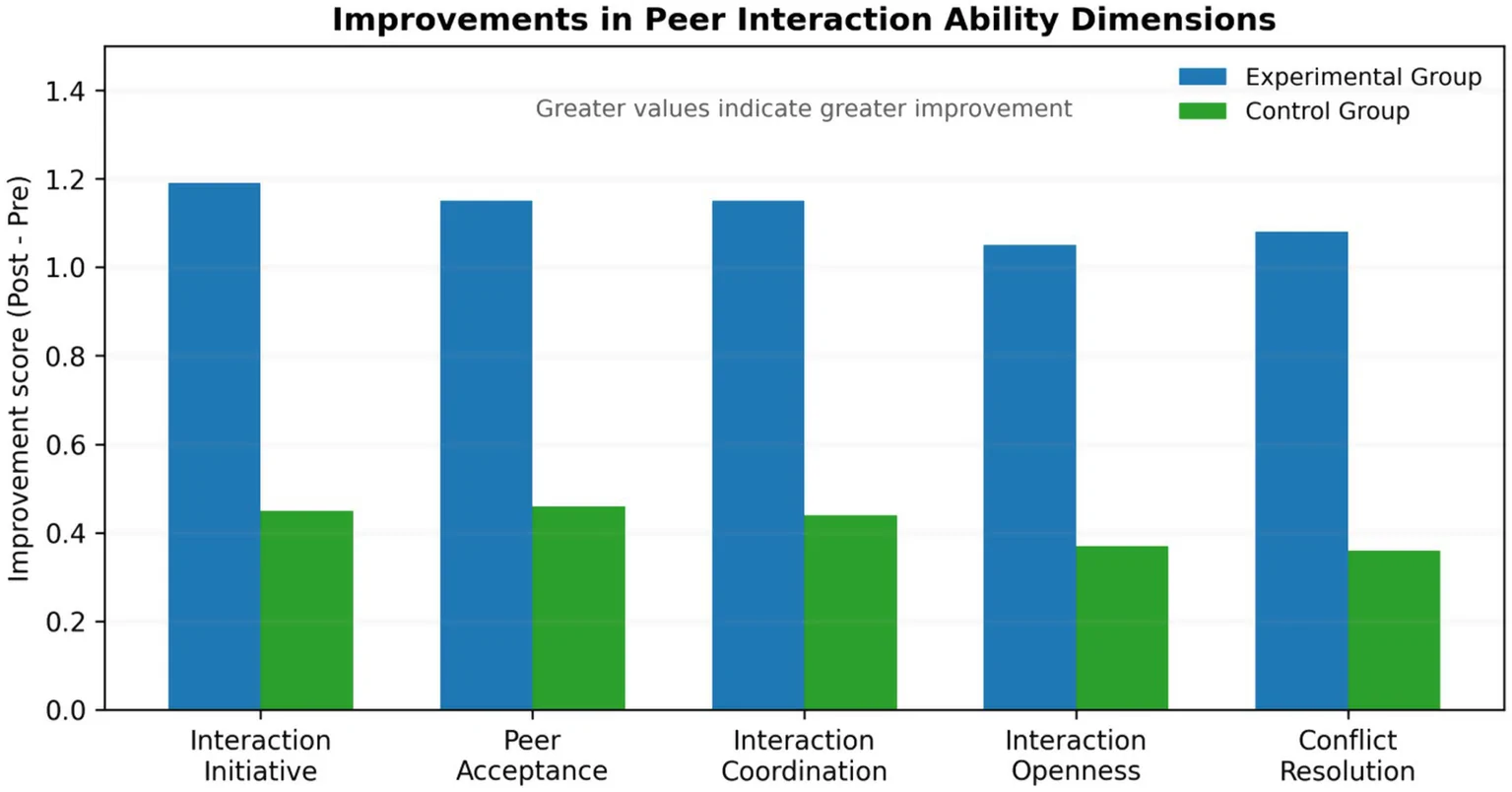
Improvements in peer interaction ability dimensions after the intervention.

### 3.4 Mixed-design ANOVA results for intervention effects

The mixed-design ANOVA demonstrated significant Group × Time interaction effects for interaction initiative (*F*(1, 78) = 57.84, *p* < 0.001, *η*^2^*p* = 0.426), peer acceptance (*F*(1, 78) = 62.17, *p* < 0.001, *η*^2^*p* = 0.443), interaction coordination (*F*(1, 78) = 55.93, *p* < 0.001, *η*^2^*p* = 0.418), interaction openness (*F*(1, 78) = 49.86, *p* < 0.001, *η*^2^*p* = 0.390), and conflict resolution ability (*F*(1, 78) = 51.72, *p* < 0.001, *η*^2^*p* = 0.399). These analyses therefore focus on the five explicitly defined peer-interaction dimensions rather than an additional overall composite.

The control group experienced modest improvements on a few scales; this could relate to overall physical activity involvement. The experimental group had greater increases in both affective/interpersonal aspects and thus demonstrated a higher level of effect than the control group from engaging in the cooperative-tactical training aspect of volleyball (Table 4).

**Table 4:** Mixed-design ANOVA results for intervention effects on social anxiety and peer interaction ability.

Outcome	Group × Time *F*(1, 78)	*p*-value	Partial *η*^2^
Total Social Anxiety Score	68.42	<0.001	0.467
Fear of Negative Evaluation	54.76	<0.001	0.413
Social Avoidance and Distress	61.35	<0.001	0.440
Interaction Initiative	57.84	<0.001	0.426
Peer Acceptance	62.17	<0.001	0.443
Interaction Coordination	55.93	<0.001	0.418
Interaction Openness	49.86	<0.001	0.390
Conflict Resolution Ability	51.72	<0.001	0.399

### 3.5 Safety and intervention compliance

There were no major side effects throughout the twelve week trial. Some children said they had some fatigue and minor muscle soreness for a short time after doing the workout. The soreness stopped after they rested and relaxed for a while after the workout. None of the participants suffered from major athletic injuries, emotional issues due to discomfort, or withdrew as a result of discomfort.

Children and their parents generally found the program to be very acceptable. This suggests that Collective Volleyball Cooperative Tactical Training can be safely conducted with school-aged children as long as it is done under the direction of a qualified coach or other professional.

## 4 Discussion

This research investigated the effects of a 12-week collective volleyball cooperative tactical training program with respect to social anxiety and ability to interact with peers by children who are 8–12 years old. Results indicated participants in this cooperative tactical volleyball intervention experienced a larger decline in their levels of social anxiety, and also experienced an improved level of peer interaction abilities compared to the participants in the standard physical education control group. Therefore, these results indicate that cooperative sport team training can be used as a viable school based method to promote the psychosocial growth of children.

Another major outcome from this study revealed that a large reduction in children’s social anxiety occurred when participating in collective volleyball cooperative tactical training. A child who has social anxiety may exhibit an excessive concern for being negatively evaluated by others; avoid interactions with other people due to fear of rejection or embarrassment; experience discomfort while communicating with other people socially. The children involved in the current program did not have to verbally discuss their thoughts or feelings with each other. They participated with other children on specific volleyball related tasks including passing, receiving, team work in small groups, and coordinating team tactics. Since these activities are based upon working together to accomplish a task and are less likely to be perceived as a source of judgment by peers compared to direct conversation or interaction, these types of activities can assist children to become accustomed to group participation (5–8, 14, 15).

The reduction of fear of peer disapproval can relate to the structure of a co-op setting. Traditionally, competition among young players is associated with a concern for the evaluation of their peers. In contrast, the volleyball tactics and team building that occurred in this study were based on cooperation; all children had to work together as a unit toward an end goal. The focus was on working collectively, sharing knowledge and expertise, and supporting one another. Children who experience repeated success while working within a positive supportive environment are less likely to have fears regarding how they will be perceived by other children (16, 18, 26, 27).

Children that have social anxiety will likely avoid new or unknown social interactions as a result of the lack of confidence when communicating interpersonally. As a child continues through volleyball cooperative training, they are exposed to multiple, consistent, and predictable ways to interact with peers during team-based activities such as calling for the ball, reminding teammates of actions needed, moving to assist other players on their team, and responding to the needs of the group. While interacting within this setting is active, it is generally less stressful than many other forms of social interaction. Through repeated, successful experiences with peers, children can become accustomed to being around others and reduce their tendency to avoid social interactions.

One of the primary results obtained from this study is an increase in students’ ability to interact positively with their peers as a result of participating in Volleyball Cooperative Tactical Training. As compared to the Control Group, the Experimental Group demonstrated a significantly larger gain in taking the initiative to engage in peer interactions, being accepted by their peers, coordinating and facilitating peer interactions, expressing openness and receptivity during peer interactions, and resolving conflicts with other peers. All of these elements relate to developing students’ ability to form and sustain peer relationships in the classroom setting. In addition to reducing the occurrence of negative social–emotional responses, the data indicates that Volleyball Cooperative Tactical Training can help foster positive interpersonal behaviors among students.

The development of an active interaction initiative among peers in this study could be due to the nature of the volleyball training. In all team based aspects of volleyball such as during cooperative passing and receiving or when children are completing various tactical assignments, they will need to make their intentions known verbally while asking for assistance from their teammates. This provides multiple opportunities for verbal communication; thus providing numerous opportunities for children to become increasingly comfortable initiating interactions. Unlike most forms of instructional content that teach social skills within a classroom setting, the nature of volleyball provides many instances where the children can interact with one another on a daily basis. These interactive experiences present the opportunity for children to demonstrate communication and complete task(s) by communicating—peer acceptance improved following intervention. The improvement in peer acceptance likely resulted from the team-based nature of the cooperative volleyball activities allowing children to feel both included and valued as part of a cohesive unit. Children who participate with their team are often rewarded through peer recognition of their contributions toward shared successes. By focusing on how the entire team works together toward common goals, this type of intervention promotes cooperation over competition and encourages inclusion of all participants (3, 4, 19, 20).

Children demonstrated an improvement in their interaction coordination and conflict management abilities. Children have to work together as a team when they participate in volleyball cooperative tactical training; each child has to be able to coordinate his/her own movement, make decisions about who will do what, adhere to the rules of the game, and adapt to the needs of the team. When participating in this type of training, there is always going to be some form of disagreement or mistake that occurs. However, by guiding them in problem solving using verbal communication and teamwork, these types of activities can lead children to develop more adaptable ways for interacting with others and manage conflicts.

When compared to standard physical education programs, a cooperative volleyball program that is designed specifically as a team will have a much more defined focus on how psychological elements are developed within teams. A standard physical education program may offer students an opportunity to engage in physical activity; may help improve their mood or overall physical fitness; however, it typically lacks intentional team communication, the use of cooperative strategies/tactics, mutual support among teammates, or a structured approach for developing groups based on roles. As such, when compared to the non-structured nature of a standard physical education program, this study was able to create a highly structured environment where all four aspects were built into the structure of the team’s training program. Therefore, it would be expected that the team participating in the cooperative volleyball program would show significantly higher gains than the team in the standard physical education program.

The theory of social learning states that a child develops their own social behavior based on what they see, do and experience with others. Social Learning Theory states that children develop their social skills by observing other people’s social behaviors, by copying or modeling those behaviors, by participating in those behaviors, and receiving feedback from the people around them. When working together during volleyball cooperative team tactics, children are able to observe positive peer interactions, copy good teamwork and communications, participate in team efforts and immediately get feedback from coaches and peers. Children will continue to repeat these experiences as they develop their ability to interact socially in an appropriate manner. Children who have success with their teammates, especially when it comes to being able to work together, will be more confident interacting with peers which will reduce their tendency to avoid social situations (26, 27).

From a school-based physical education context, this study indicated that using sport training as a means of promoting not only physical, but psychological and social development. Volleyball is commonly provided within schools, has minimal equipment requirements, and allows for adaptation to both physical and cognitive characteristics of children. With organization centered on cooperation and communication with peers during volleyball training, children’s social confidence and overall quality of peer relationships may be positively enhanced (21, 22).

The conclusions from this research will be beneficial for application. With respect to providing interventions to school aged children that are either mildly socially anxious or lack sufficient peer interaction experiences; there can be limitations as to how willing children are to engage psychologically. Sport based intervention is often an interactive way to playfully and non-stigmatized way for children to develop their interpersonal skills. Incorporating team based activities such as volleyball training into school PE programs could provide a natural and accepting environment for developing these skills. Additionally, it could be used to support the integration of body–mind type education into school environments.

The first limitation is that our sample size was somewhat limited. While we were able to use an appropriate number of participants (*n* = 80) for the intervention we have implemented, larger numbers will likely be needed to make our results generalize to a broader population. The second limitation is that our program was a 12-week one, and we did not collect data after those 12-weeks. Thus, it is difficult to say if the positive outcomes noted in this study would be sustained beyond completion of the program. The third limitation is that most of the information collected in this study came from self-report questionnaires. We suggest that in future studies, researchers could gather additional information about the social aspects of their subjects through teacher ratings, behavioral observations, parent reports or even qualitative interviews. A fourth limitation concerns expectancy and social desirability bias. Because the physical nature of the intervention made participant blinding impossible, children were aware of whether they were receiving cooperative volleyball training or standard physical education. Although questionnaires were administered anonymously in a quiet classroom by trained research assistants who were not involved in intervention delivery, this awareness may have influenced post-intervention self-reports, particularly if participants expected improvement or wished to respond favorably after taking part in the novel cooperative program. Future studies should combine self-report measures with blinded independent observer ratings, teacher or parent reports, and behavioral indicators of peer interaction. A fifth limitation concerns measurement validation. The study-specific 25-item peer-interaction questionnaire was informed by the broader ICQ framework and by published Chinese adolescent/youth adaptations, including psychometric validation of the Chinese ICQ-15 and a published 25-item shortened ICQ precedent (23–25). However, the exact item set and domain labels used in the present study are not identical to those published instruments. Accordingly, the external validation evidence for ICQ-based measures cannot be assumed to validate the exact questionnaire used here. Although internal consistency was high in the present sample (Cronbach’s *α* = 0.91), internal consistency alone does not establish construct validity. Future studies should independently evaluate the factorial structure, convergent validity, test–retest reliability, and measurement invariance of the present form, or replicate the findings using a published externally validated peer-interaction instrument.

The study has shown some evidence that the training of volleyball cooperative tactics can help to reduce social anxiety and improve a child’s ability to interact with their peers. The program is especially important because it teaches the importance of working together, communicating, and achieving common goals. The most significant element of the training is not necessarily just getting children involved in an activity; it is how the training allows them to work cooperatively as a group.

## 5 Conclusion

This study found that a 12-week collective volleyball cooperative tactical training program was associated with reduced social anxiety and improved peer interaction ability in children aged 8–12 years. Compared with the standard physical education control group, the experimental group showed greater reductions in SASC total scores, fear of negative evaluation, and social avoidance and distress, together with greater improvements in interaction initiative, peer acceptance, interaction coordination, interaction openness, and conflict resolution ability.

The intervention offered children a supportive and natural opportunity for social interaction with their peers by way of working together in teams on offense and defense through such activities as cooperative passing; working in groups to complete both small group objectives and large group objectives; creating opportunities for children to coordinate tactics and work as a team.

Collective Volleyball Cooperative Tactical Training as a School-Based Model to Promote Student Mental Health (Social Development) through Physical Education can be safely implemented into schools. This type of training could assist in providing teachers and students alike with an opportunity to integrate child mental health promotion and social development education in their physical education programs. Future research will need to assess the collective volleyball cooperative tactical training’s longitudinal impact on student mental health and social development, as well as assess if it is effective across various educational environments and age groups.

## Data Availability

The raw data supporting the conclusions of this article will be made available by the authors, without undue reservation.
