# Supplementary material for: Effects of collective volleyball cooperative tactical training on social anxiety and peer interaction ability in school-aged children: a controlled intervention study
Source: Front Public Health. 2026 Oct 28;14:1952919. doi: 10.3389/fpubh.2026.1952919 (PMC13751365; doi:10.3389/fpubh.2026.1952919)
Supplement: Supplementary file 1 [file Table_1.DOCX]

Supplementary Materials

# Supplementary Table S1. Weekly Volleyball Cooperative Tactical Training Schedule

| Stage | Weeks | Main Content | Psychosocial Objective |
| --- | --- | --- | --- |
| Adaptation stage | Weeks 1–2 | Basic volleyball skills, group familiarity, cooperative games | Building peer trust and reducing interaction anxiety |
| Cooperative tactical training | Weeks 3–10 | Team positioning, cooperative tactics, problem-solving drills, group tasks | Enhancing communication, coordination, and peer interaction |
| Consolidation stage | Weeks 11–12 | Integrated tactical games and reflective teamwork activities | Maintaining social confidence and cooperative behaviors |

# Supplementary Table S2. Children's Peer Interaction Ability Scale

The Children's Peer Interaction Ability Scale used in this study contains 25 items across five dimensions: interaction initiative, peer acceptance, interaction coordination, interaction openness, and conflict resolution ability. Each dimension contains five items. The assessed domains are consistent with established child peer-relations and youth-sport peer-functioning literature cited in the main manuscript (references 5, 7, 27, and 29). Responses are rated on a 5-point Likert scale from 1 (strongly disagree) to 5 (strongly agree), with higher scores indicating stronger peer interaction ability. Each dimension score is calculated as the arithmetic mean of its five corresponding items. In the revised manuscript, the five dimension scores are reported and analyzed separately. An additional Overall Peer Interaction Ability composite is not reported because the available scoring documentation does not establish a distinct, independently interpretable overall scoring algorithm beyond the five equally sized dimensions. Internal consistency for the complete 25-item instrument in the present sample was Cronbach's α = 0.91.

# Supplementary Table S3. Randomization and Allocation Concealment Procedure

After baseline assessment, participants were randomly assigned to the experimental or control group using a computer-generated random sequence. An independent researcher who was not involved in intervention delivery generated the allocation sequence. Group assignments were placed in sealed opaque envelopes prepared by that independent researcher. The envelopes remained closed until baseline assessment was complete and were opened only when the participant's group assignment was implemented, thereby maintaining allocation concealment before baseline data collection.
